# The Alteration of T-Cell Heterogeneity and PD-L1 Colocalization During dMMR Colorectal Cancer Progression Defined by Multiplex Immunohistochemistry

**DOI:** 10.3389/fonc.2022.867658

**Published:** 2022-05-20

**Authors:** Hongkai Yan, Yaqi Li, Xiaoyu Wang, Juanjuan Qian, Midie Xu, Junjie Peng, Dan Huang

**Affiliations:** ^1^Department of Colorectal Surgery, Fudan University Shanghai Cancer Center, Shanghai, China; ^2^Department of Oncology, Shanghai Medical College, Fudan University, Shanghai, China; ^3^Laboratory of Immunology and Virology, Experiment Center for Science and Technology, Shanghai University of Traditional Chinese Medicine, Shanghai, China; ^4^Department of Medicine, Genecast Biotechnology, Beijing, China; ^5^Department of Pathology, Fudan University Shanghai Cancer Center, Shanghai, China; ^6^Institute of Pathology, Fudan University, Shanghai, China

**Keywords:** tumor microenvironment, PD-L1 co-localization, mismatch repair-deficient, colorectal cancer, tumor progression, multiplex immunohistochemistry

## Abstract

**Background:**

Immune checkpoint inhibitors (ICIs) are quickly becoming key instruments in the treatment of mismatch repair-deficient (dMMR) colorectal cancers (CRCs). Despite their clinical value, ICIs have several limitations associated with their use. Only approximately 15% of all CRCs have a dMMR status, and the overall response rate of ICIs is approximately 40%. The mechanism of ICI resistance is not clear, and its study is limited by the lack of information available on the characterization of the immune microenvironment during the progression from early- to advanced-stage dMMR CRC.

**Methods:**

We used multiplex immunohistochemistry (mIHC) with two panels, each containing five markers, to simultaneously analyze the proportions of immune microenvironment constituents in 59 patients with advanced-stage dMMR CRC and 24 patients with early-stage dMMR CRC. We detected immune cell–associated signatures in the epithelial and stromal regions and evaluated the predictive value of these immune molecules. Student’s t-tests, Mann–Whitney U tests, Cox proportional hazards regression modeling, univariate Cox modeling, and Kaplan–Meier estimation were used to analyze immune cell proportions and survival data.

**Results:**

We observed significantly higher proportions of CD8+ cytotoxic T cells (CD8+) (*p* = 0.001), CD8+ memory T cells (CD8+CD45RO+) (*p* = 0.032), and CD4+ regulatory T cells (CD4+FOXP3+) (*p* = 0.011) in the advanced-stage dMMR CRCs than in the early-stage dMMR CRCs. Furthermore, CD3+ T cells with PD-L1 colocalization (CD3+PD-L1+) (*p* = 0.043) and CD8+ T cells with PD-L1 colocalization (CD8+PD-L1+) (*p* = 0.005) were consistently more numerous in patients in the advanced stage than those in the early stage. Our analyses revealed that a high proportion of CD3+PD-1+ T cells was an independent prognostic factor of overall survival (OS) [hazard ratios (HR) = 9.6, *p* < 0.001] and disease-free survival (DFS) (HR = 3.7, *p* = 0.010) in patients in the advanced stage.

**Conclusion:**

High numbers of CD8+ cytotoxic T cells and CD8+ memory T cells, which usually represent a cytotoxic function of the adaptive immune system and possibly enhanced inhibition factors, such as CD4+ regulatory T cells and PD-L1 colocalized T cells, were associated with the transformation of the immune microenvironment from the early stage to the advanced stage in dMMR CRCs. Furthermore, CD3+PD-1+ T cells are a prognostic factor for patients with dMMR.

## Introduction

Colorectal cancer (CRC) is a genetically heterogeneous disease, and patients with CRC show widely disparate outcomes. Recently, the significant immunotherapeutic benefit of immune checkpoint inhibitors (ICIs) in CRCs has been reported in patients with dMMR (mismatch repair-deficient) CRC (1–3). dMMR tumors may occur *via* several mechanisms. In Lynch syndrome CRC, the underlying mechanism is usually a germline mutation of one of the four (MLH1, MSH2, MSH6, and PMS2) MMR genes, leading to microsatellite instability (MSI). In contrast, in sporadic CRC, dMMR is primarily caused by epigenetic silencing through CpG methylation of the MLH1 gene promoter, among other causes (4). dMMR tumors are often accompanied by a higher density of tumor-infiltrating lymphocytes (TILs) and more cytotoxic T cells than mismatch repair-proficient (pMMR) tumors (5, 6). The most likely explanation for the vital benefit of immunotherapy is the high levels of neoantigens, which trigger T-cell response to tumor cells in patients with dMMR (7, 8).

The dMMR status of CRCs varies across different stages of the disease. Overall, approximately 15% of all CRCs have dMMR status, but dMMR status becomes less common in later stages: approximately 20% in stage II, 12% in stage III, and only 2% to 4% in stage IV (9, 10). Previous studies have proposed that this phenomenon is associated with a decrease in immune surveillance (11). Patients with early-stage CRC with dMMR have a significantly better prognosis and longer survival than pMMR patients; however, in contrast with early-stage CRCs, dMMR predicts a significantly worse prognosis in advanced-stage CRCs (12). Considering the relationship between T cells and the dMMR status, it is hypothesized that the heterogeneity of T cells contributes significantly to the transformation of the immune microenvironment from the early stage to the advanced stage in dMMR CRCs.

The response rate to immunotherapy differs in early- and advanced-stage dMMR tumors. Immunotherapy may be highly effective in patients with early-stage dMMR CRC. In an exploratory NICHE study (NCT03026140) of neoadjuvant treatment with nivolumab (anti–PD-1 antibody) and ipilimumab (anti–CTLA-4 antibody) in patients with dMMR, the pathological response rate was 100% in 20 patients with dMMR (13). However, in advanced-stage dMMR tumors, a meta-analysis of 939 dMMR/MSI-H (MSI-high) patients pretreated with ICIs from 14 studies indicated a pooled overall response rate of 41.5% (14). In addition, certain biomarkers can predict the efficacy of ICIs, such as the Immunoscore, which measures TILs in the core and invasive margins of a tumor (15), the tumor mutation burden (16), and the T-cell phenotype (17). Although the mechanisms of immunotherapy resistance are unclear, some possible mechanisms have been proposed, such as the infiltration of CD8+ T cells or FOXP3+ cells (18, 19), the limited repertoire of cytotoxic T cells (20), and the absence of memory T cells (21). T-cell immunotherapies have shown great promise in patients with advanced cancer and have revolutionized treatment (22). Therefore, we presumed that the heterogeneity of T cells played a crucial role in ICI response at different stages of dMMR tumors. Among all the immune checkpoints, we only explored PD-1/PD-L1 because this checkpoint has been well studied, and a considerable amount of literature on it has been published. The PD-1/PD-L1 inhibitors are the most widely used ICIs. Current research is investigating opportunities for their use in every stage of CRC (23). Although this area has been the focus of much research, knowledge of TILs between early-stage and advanced-stage dMMR CRC is limited.

In the present study, we sought to study the distribution of individual immune cells subsets, especially T cells, in early- and advanced-stage dMMR CRCs and to reveal their effect on patient survival. We designed two multiplex immunohistochemistry (mIHC) panels consisting of immune cell markers. The following T-cell markers were considered: CD3, which is highly expressed by T cells; CD4, which is a marker of T helper cells; CD8, which is highly expressed by cytotoxic T cells; CD8+CD45RO+, which is a memory cytotoxic T-cell marker; CD4+FOXP3+, which is a marker of CD4+ regulatory cells; CD68, which is a pan-macrophage marker; CD68+CD163−, which represents M1-like macrophages; CD68+CD163+, which is a marker of M2-like macrophages; and PD-1 and PD-L1, which are immune checkpoint markers (24–28). The simultaneous quantification of various markers in both epithelial regions and stromal regions profiles the immune characteristics of dMMR CRCs across tumor stages and may assist in the identification of potential prognostic markers.

## Materials and Methods

### Patient Selection

The Ethics Committee of Fudan University Shanghai Cancer Center approved this study. Paraffin-embedded tissue samples from patients with dMMR CRC (n = 83) diagnosed between 2013 and 2019 were collected, and patients were staged according to the American Joint Committee on Cancer staging system (eight edition). Patients in the early stage included patients with stage I or II cancer, whereas patients in the advanced stage included patients with stage III or IV cancer. We obtained 40 fresh specimens from the Department of Colorectal Surgery between 2019 and 2020. None of the patients were treated with radiation therapy, chemotherapy, or immunotherapy before tumor resection. Clinical patient information (e.g., sex and age) was collected from patient clinical records. Information regarding tumor location, size, histological parameters, and vascular and perineural invasion were obtained from pathological reports. All cases that lacked the expression of MLH1, MSH2, MSH6, or PMS2 were histologically confirmed as dMMR.

### Multiplex Immunohistochemistry

Sections (4 μm thick) were cut from formalin-fixed, paraffin-embedded (FFPE) CRC tissue and control tonsil tissue. The slides were dewaxed in xylene, rehydrated, and rinsed in graded ethanol solutions and tap water. Before the sections were boiled in Tris-Ethylene Diamine Tetraacetie Acid (EDTA) buffer (pH 9; 643901; Klinipath, Duiven, The Netherlands) for antigen retrieval and microwave treatment (MWT), an antibody diluent/block (72424205; PerkinElmer, Waltham, MA, USA) was applied to block endogenous peroxidase. Information on the primary antibodies and the corresponding fluorophores is provided in **Supplementary Table 1**, including two panels with five primary antibodies each. One antigen required one round of labeling, including primary antibody incubation, secondary antibody incubation, and tyramide signal amplification (TSA) visualization, followed by labeling of the subsequent antibody. After incubation with the primary antibody for 1 h at room temperature, Opal Polymer HRP Ms+Rb (2414515; PerkinElmer, Waltham, MA, USA) was added and incubated at 37°C for 10 min. TSA visualization was performed with the Opal 7-Color IHC Kit (NEL797B001KT; PerkinElmer, Waltham, MA, USA) containing the fluorophores DAPI (4,6-diamidino-2-phenylindole; Thermo Scientific, Rockford, IL, USA) and the TSA Coumarin system (NEL703001KT; PerkinElmer, Waltham, MA, USA). MWT was performed to remove the antibody-TSA complex with Tris-EDTA buffer (pH 9). TSA single-stained slides were finished with MWT, counterstained with DAPI for 5 min, and enclosed in Antifade mounting medium (I0052; NobleRyder, Beijing, China).

### Slide Analysis

Multiplexed and single-color control slides were scanned at an absolute magnification of ×200 using the PerkinElmer Vectra automated multispectral microscope (PerkinElmer, Inc., Hopkinton, MA, US). Representative fields from the single-color slides were imaged, and the inForm image analysis software (version 2.1, PerkinElmer) was used to generate a spectral library for unmixing. Index cases were stained using the multiplex method and then imaged. Channels were unmixed using the spectral library. All settings were saved within an algorithm to allow for batch analysis of multiple original multispectral images of the same tissue (29).

### Quantification of Immune Cells and Phenotyping

On the basis of the identification of the DAPI-stained nuclear morphological features, the numbers of immune cells in each image were scored as percent cellularity (number of positive cells/number of nucleated cells, expressed as a percentage). Five representative fields at ×200 magnification of tissue area were selected. The densities of immune cells in the tumor epithelial and stromal regions were segmented as a whole and separately by the pathologist, and they were described as one of three arrays: “total”, “epithelial”, or “stromal”. Immune variables were classified on the basis of the patterns of fluorochrome intensity.

### Follow-Up

The patients were monitored every 3 to 6 months for the first 3 years and every 6 to 12 months thereafter. Subsequent evaluations, including digital rectal examinations and serum carcinoembryonic antigen (CEA) tests, were performed at each follow-up. Radiological studies and colonoscopies were performed annually or when clinically indicated. All surviving patients were contacted between June and August 2019 *via* registered mail or telephone.

### Statistical Analyses

Statistical analyses were performed using the SPSS 25.0 software (IBM Corp., Chicago, IL, USA) and GraphPad Prism 7 software (GraphPad Software Inc., San Diego, CA, USA). Differences in the immune cell proportions in the subgroups were analyzed using the t-test (normal distribution) or the Mann–Whitney U test (non-normal distribution), as appropriate. Radar plots were created to show the percentage of immune cells obtained by multiple IHC analyses of the total number of cells in each tissue segmentation. The proportion of immune cells was ranked to obtain the percentile, from lowest to highest, in the whole queue. For immune cell subsets in each group, the average percentile was obtained and graphed on a radar plot to show the trends between different groups. A Cox proportional hazards regression model was used to assess the hazard ratios (HRs), 95% confidence intervals (CIs), and *p*-values in univariate and multivariate analyses. Each immune variable was analyzed as a continuous variable with regard to overall survival (OS) and disease-free survival (DFS) in the univariate Cox models using a log-rank test or Wald test. Variables for which *p* < 0.10 after adjusting for common clinicopathological parameters were also included in the multivariate analysis. Survival curves were plotted for each variable using the Kaplan–Meier method and compared using the log-rank test. A *p-*value <  0.05 was considered statistically significant. All *p-*values corresponded to two-sided statistical tests.

### Ethics Approval and Consent to Participate

This study was evaluated and approved by the Ethics Committee of Fudan University Shanghai Cancer Center (FUSCC), and written informed consent was obtained from all participants or their appropriate surrogates.

## Results

### Patient Characteristics

We included 83 patients with CRC with known dMMR status in our study, 59 with advanced-stage tumors, and 24 with early-stage tumors. Clinicopathological characteristics are presented in **Supplementary Table 2**. The sex ratio was close to 2:1 (male:female, 39:20) in the advanced-stage patient group and nearly 1:1 (male:female, 13:11) in the early-stage patient group. All dMMR CRCs were predominantly located in the right colon (56% in patients in the advanced stage and 67% in patients in the early stage) and were poorly to moderately differentiated. Mucinous adenocarcinoma occurred more frequently in the patients in the early stage (42%) than in the patients in the advanced stage (29%). Deficient expression of the MMR protein was distinct in each stage: MSH2 and MSH6 expression loss was more common in patients in the advanced stage, and only MSH6 expression loss was commonly detected in patients in the early stage.

### Immune Microenvironment of dMMR CRC

**Figure 1:**
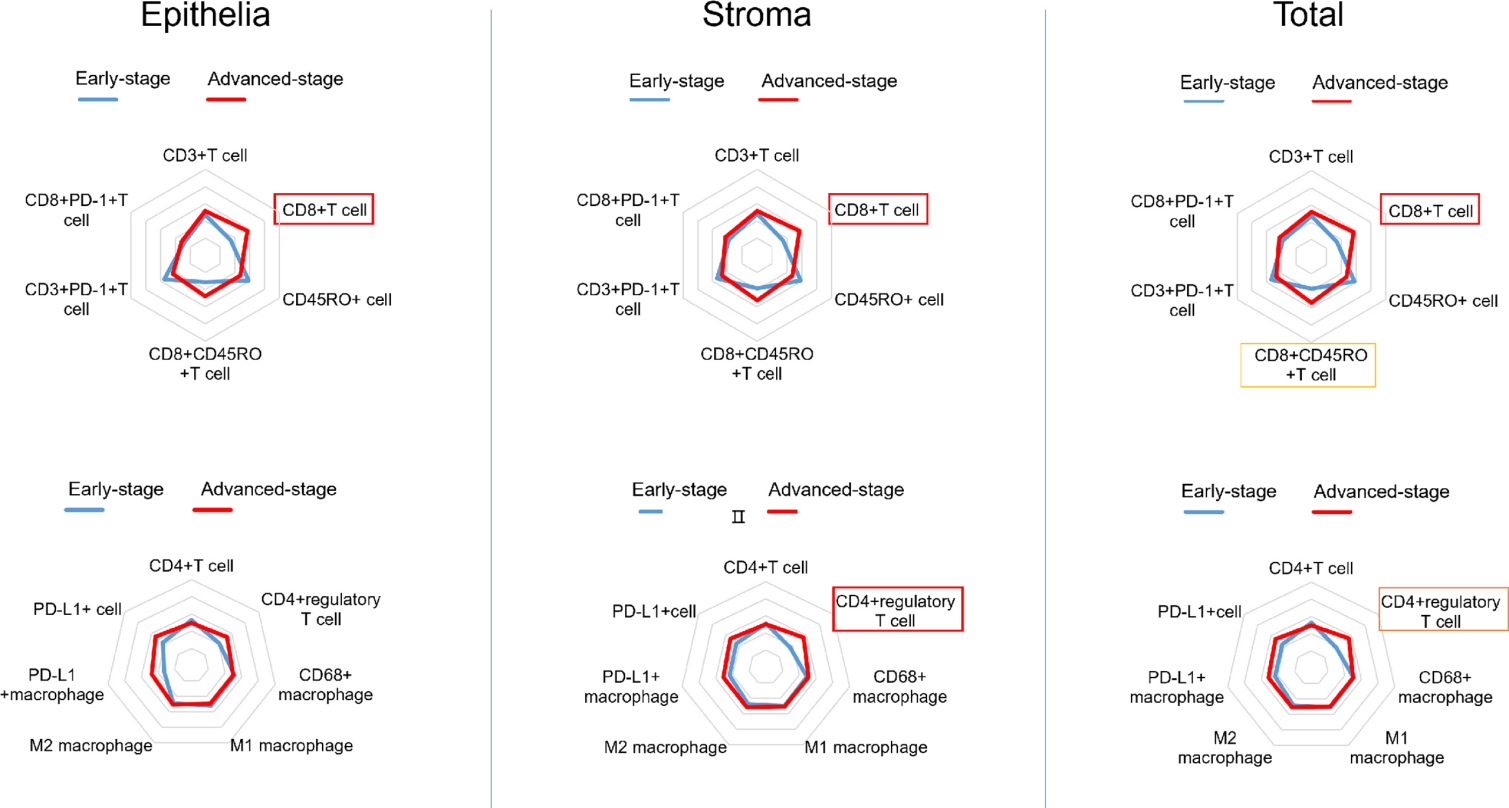
Radar plots showing the average percentile of each immune cell subset in dMMR CRCs (early stage vs. advanced stage). The density of immune cells was segmented as a whole and separately and analyzed in three arrays: epithelial regions (left), stromal regions (middle), and total regions (right). The frames indicate whether each variable is statistically significant (*p* < 0.05).

**Figure 2:**
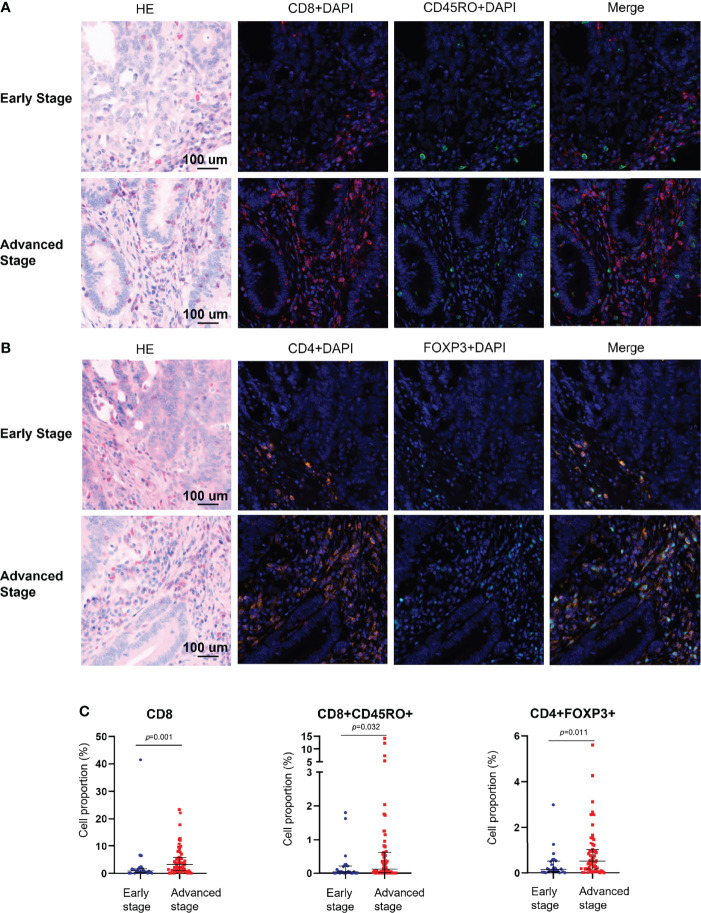
Multiplex immunohistochemical analysis of the immune composition of early-stage and advanced-stage dMMR tumors. **(A)** Representative images of CD8+ and CD45RO+ cells. **(B)** Representative images of CD4+ and FOXP3+ cells. **(C)** Pairwise comparison of the densities of intraepithelial immune cells between early-stage tumors and advanced-stage tumors. Nuclei (DAPI, blue), CD8 (cytoplasm, Opal 690, red), CD45RO (cytoplasm, Opal 520, green), CD4 (cytoplasm, Opal 690, orange), and Foxp3 (membrane, Opal 570, indigo).

The mIHC panels were used to detect differences in the composition of immune cells within tumor stages. Comparisons of the proportions of immune cells between the early and advanced stages were graphed for epithelial regions, stromal regions, and total regions in radar plots (**Figure 1**). T-cell composition was significantly different between the epithelial and stromal regions (**Figure 1**). The proportion of CD8+ cytotoxic T cells in all regions was 2.99% in early-stage tumors and 4.62% in advanced-stage tumors (*p* = 0.001). Furthermore, 0.22% CD8+ memory T cells (CD8+CD45RO+ T cells) were observed in the total regions in early-stage tumors, whereas 1.02% were observed in advanced-stage tumors (*p* = 0.032). The total regions exhibited more CD4+ regulatory T cells (CD4+FOXP3+ T cells) in patients in the advanced stage (0.85%) than in patients in the early stage (0.38%) (*p* = 0.011) (**Figure 2**), and a similar composition (0.32% in patients in the advanced stage and 0.16% in patients in the early stage) was observed in stromal regions (*p* = 0.007). No significant differences were detected in the levels of macrophages in different stages of dMMR tumors (**Figure 1**). Comparisons between immune cell proportions in epithelial regions and stromal regions are illustrated in radar graphs (**Supplementary Figure 1**).

### PD-L1 and PD-1 Expression in Tumor Epithelial and Stromal Regions

**Figure 3:**
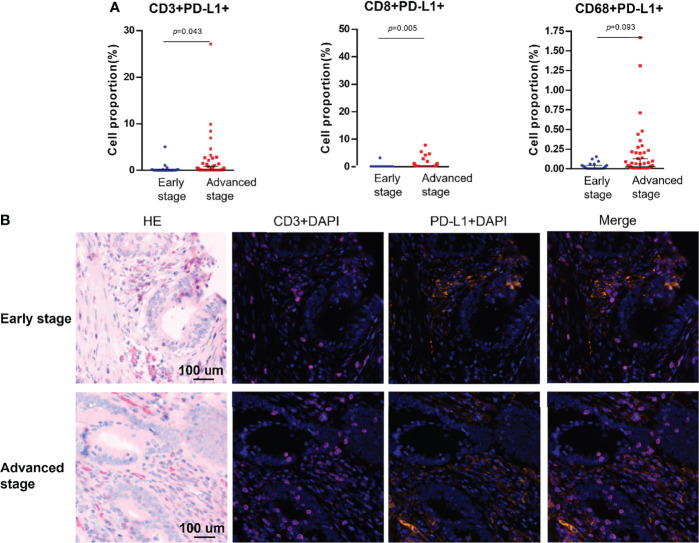
Colocalization of PD-L1 with immune cells markers using multiplex immunohistochemistry. **(A)** Pairwise comparisons of PD-L1+ immune cells in the tumor epithelium in early-stage and advanced-stage tumors. **(B)** Representative images of PD-L1+CD3+ cells in dMMR CRCs by stage. Nuclei (DAPI, blue), CD3 (cytoplasm, Opal 650, purple), and PD-L1 (membrane, Opal 690, orange).

PD-L1 and PD-1 were expressed on the tumors, but no difference in expression was observed between stages (**Table 1**). Among the analyzed immune cell proportions, PD-L1+ cells were more frequently observed in advanced-stage tumors than in early-stage tumors in epithelial regions (5.08% and 2.74%, respectively), such as CD3+PD-L1+ T cells (colocalization of PD-L1 on CD3+ T cells) (*p* = 0.043), CD8+PD-L1+ T cells (colocalization of PD-L1 on CD8+ T cells) (*p* = 0.005), and PD-L1+ macrophages (colocalization of PD-L1 on CD68+ T cells) (*p* = 0.093) (**Figure 3**). Furthermore, colocalization of PD-L1 on CD8+ T cells was abundant in the associated stromal regions of advanced dMMR tumors (*p* = 0.007) (**Table 1**). However, in the epithelial regions, the proportion of PD-1+ cells was significantly higher in early-stage tumors than in advanced-stage tumors (**Table 1**).

**Table 1:** Immune composition of dMMR CRCs in epithelial and stromal regions across tumor stages.

Variable	Epithelial Region			Stromal Region		
	Early Stage (%)	Advanced Stage (%)	*p-*value	Early Stage (%)	Advanced Stage (%)	*p-*value
PD-L1+ epithelium	2.25	4.89	0.131	–	–	–
PD-L1+ stromal cells	–	–	–	3.45	5.50	0.200
CD3+PD-L1+ cells	0.32	1.39	0.043	0.41	1.56	0.105
CD8+PD-L1+ cells	0.14	0.60	0.005	0.08	0.80	0.007
CD68+PD-L1+ cells	0.03	0.13	0.093	0.22	0.70	0.264
CD68-PD-L1+ cells	0.82	2.67	0.006	2.71	4.81	0.058
CD68+CD163+PD-L1+ cells	0.01	0.06	0.155	0.10	0.37	0.390
PD-1+ epithelium	1.50	0.44	0.005	–	–	–
PD-1+ stromal cells	–	–	–	3.60	2.08	0.083
CD3+PD-1+ cells	0.54	0.21	0.191	1.74	1.10	0.393
CD8+PD-1+ cells	0.07	0.04	0.794	0.30	0.22	0.675

### Prognostic Impact of Immune Cell Infiltration

**Figure 4:**
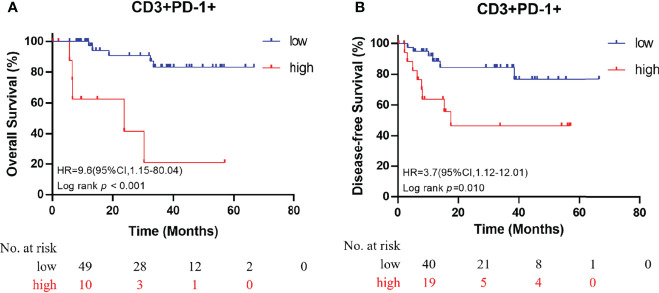
Kaplan–Meier curves for overall survival **(A)** and disease-free survival **(B)** in patients with advanced-stage dMMR CRC, according to the proportion of dual-positive CD3+PD-1+ cells.

All immune cell phenotypes and clinicopathological characteristics were entered into the Cox proportional hazards regression model. For the advanced-stage group, the univariate analysis revealed that a high percentage of CD3+PD-1+ T cells was a prognostic factor for OS (HR = 2.87, 95% CI = 1.57–5.25, *p* = 0.001, **Table 2**). When analyzed in a multivariate model adjusted for this variable, a high proportion of CD3+PD-1+ T cells remained an independent predictor for poor OS (HR = 3.22, 95% CI = 1.46–7.09, *p* = 0.004, **Table 3**). CD3+PD-1+ T cells (HR = 2.13, 95% CI = 1.25–3.64, *p* = 0.006) and CD8+PD-1+ T cells (HR = 2.92, 95% CI = 1.08–1.84, *p* = 0.034) were prognostic factors for DFS in the univariate analysis, but the multivariate survival analyses showed that only CD3+PD-1+ T cells were an independent prognostic factor for DFS (HR = 2.96, 95% CI = 1.49–5.89, *p* = 0.002, **Table 4**). The Kaplan–Meier survival curves for OS revealed that a higher percentage of CD3+PD-1+ T cells (HR = 9.6, 95% CI = 1.15–80.04, log rank *p* < 0.001) had poor prognostic value for patients with advanced-stage CRC with a known dMMR status (**Figure 4A**). The same results were obtained for the DFS survival curves (HR = 3.7, 95% CI = 1.12–12.01, log rank *p* = 0.010) (**Figure 4B**). However, the other immune cell phenotype had no prognostic value in early-stage dMMR (data not shown).

**Table 2:** Univariate analysis for overall survival and disease-free survival in advanced-stage dMMR CRCs.

Phenotype	Variable	OS		DFS	
		HR (95%CI)	*p-*value	HR (95% CI)	*p-*value
T cell	CD3+	1.03 (0.97–1.1)	0.364	1.03 (0.99–1.08)	0.105
CD4+ T cell	CD4+	1.07 (0.92–1.24)	0.413	1.09 (0.96–1.22)	0.171
CD8+ T cell	CD8+	0.91 (0.74–1.12)	0.368	1.05 (0.95–1.15)	0.335
CD45RO cell	CD45RO+	1.03 (0.95–1.11)	0.489	1.03 (0.98–1.08)	0.229
Memory T cell	CD8+CD45RO+	0.81 (0.36–1.83)	0.618	1.11 (0.96–1.29)	0.169
Macrophage	CD68+	1.05 (0.91–1.22)	0.516	1 (0.86–1.16)	0.987
M1 macrophage	CD68+CD163−	1.11 (0.91–1.36)	0.299	0.98 (0.77–1.26)	0.894
M2 macrophage	CD68+CD163+	1 (0.65–1.52)	0.984	1.03 (0.74–1.43)	0.870
CD4+ regulatory T cell	CD4+FOXP3+	1.24 (0.76–2.03)	0.388	1.17 (0.72–1.91)	0.520
CD3+PD-1+ T cell	CD3+PD-1+	2.87 (1.57–5.25)	0.001	2.13 (1.25–3.64)	0.006
CD8+PD-1+ T cell	CD8+PD-1+	0.62 (0.02–17.41)	0.776	2.92 (1.08–7.88)	0.034
PD-L1+ macrophage	PD-L1+CD68+	0.18 (0.02–1.75)	0.140	0.58 (0.18–1.84)	0.351
PD-L1+ cell	PD-L1+	0.94 (0.8–1.11)	0.469	1.03 (0.97–1.09)	0.377

The p-values reported are associated with the Wald test.

**Table 3:** Multivariate analysis of overall survival in advanced-stage dMMR CRCs.

Variable		n	Events (%)	Unadjusted HR (95% CI)	*p*-value	Adjusted HR (95% CI)	*p*-value
M stage	M0	46	6 (13)	1	0.043	1	0.041
	M1	12	4 (33)	3.75 (1.04–13.44)		10.47 (1.1–99.36)	
Tumor size	≤5cm	31	7 (23)	1	0.408	1	0.524
	>5 cm	28	3 (11)	0.56 (0.15–2.18)		1.82 (0.29–11.39)	
Vascular invasion	no	27	5 (19)	1	0.912	1	0.478
	yes	31	5 (16)	0.93 (0.27–3.23)		0.57 (0.12–2.7)	
Perineural invasion	no	41	6 (15)	1	0.355	1	0.264
	yes	18	4 (22)	1.82 (0.51–6.48)		2.52 (0.5–12.71)	
LNR^*^	<0.2	38	4 (11)	1	0.054	1	0.017
	≥0.2	21	6 (29)	3.48 (0.98–12.36)		10.75 (1.52–75.77)	
CD3+PD-1+	–	–	–	2.87 (1.57–5.25)	0.001	3.22 (1.46–7.09)	0.004

LNR, Lymph node ratio between metastatic and examined lymph nodes.

**Table 4:** Multivariate analysis of disease-free survival in advanced-stage dMMR CRCs.

Variable		n	Events (%)	Unadjusted HR (95% CI)	*p*-value	Adjusted HR (95% CI)	*p*-value
Differentiation	poor	31	11 (35)	1	0.035	1	0.108
	moderate to well	26	2 (8)	0.2 (0.04–0.9)		0.27 (0.05–1.34)	
Tumor size	≤5 cm	31	9 (29)	1	0.507	1	0.997
	>5 cm	28	5 (18)	0.69 (0.23–2.06)		1 (0.28–3.54)	
Vascular invasion	no	27	5 (19)	1	0.588	1	0.79
	yes	31	8 (26)	1.36 (0.44–4.18)		1.21 (0.29–5.05)	
Perineural invasion	no	41	5 (12)	1	0.003	1	0.015
	yes	18	9 (50)	5.2 (1.73–15.59)		4.78 (1.36–16.79)	
LNR	<0.2	38	7 (18)	1	0.13	1	0.198
	≥0.2	21	7 (33)	2.25 (0.79–6.46)		2.15 (0.67–6.91)	
CD3+PD-1+	–	–	–	2.13 (1.25–3.64)	0.006	2.96 (1.49–5.89)	0.002

## Discussion

Immune cells play important roles in inflammation and tumorigenesis, changing normal colorectal tissues to tumor tissues, which, in turn, increase the immune cell imbalance in tumors. In previous research, the infiltration of 22 immune cell types in 404 CRCs and 40 adjacent non-tumorous tissues was analyzed using CIBERSORT, a deconvolution algorithm. Macrophages and CD4 memory-activated T cells were found to be infiltrated significantly more in CRCs than in normal tissues in both The Cancer Genome Atlas (TCGA) and Gene Expression Omnibus (GEO) cohorts (30). Inflammation could affect CRC tumorigenesis and modulate the polarization of cells in the TME and the corresponding cytokine milieu, causing tumor-elicited inflammation (31). In inflammation-associated tumorigenesis, chronic inflammation resulting from infections, dysregulated immune responses, or environmental factors can initiate and promote tumorigenesis through the induction of DNA damage or epigenetic changes (32–34). Oncogene activation and tumor suppressor loss sustain tumor-elicited inflammation by indirectly inducing the production of pro-inflammatory cytokines, growth factors, and chemokines that recruit inflammatory immune cells to the tumor site (35, 36).

Little is known about the immune cells that promote dMMR tumor progression. Therefore, further exploration of the immune microenvironment in dMMR tumor progression is needed. Recent advances in mIHC have enabled the simultaneous analysis of several markers within a single FFPE tissue section (29). Our present study used this novel mIHC technique with two panels to show a comparable immune microenvironment across different stages of dMMR tumors, with accurate cell discrimination and spatial information (**Supplementary Figure 1**). The immune microenvironment at different stages was plotted on the basis of the intensity of several markers of immune cells, providing evidence for a distinct spectrum of immune populations in the early and advanced stages. Researchers have shown that CD8+ cytotoxic T cells and CD8+ memory T cells are crucial to antitumor immunity (22, 37). However, we observed an unusually high proportion of CD8+ cytotoxic T cells and CD8+ memory T cells in patients with advanced dMMR. Researchers have demonstrated that when infiltrating cancer tissues, CD8+ T cells are generally in dysfunctional states (38). Our findings support two possible explanations for these dysfunctional states.

First, some researchers have outlined that the binding of PD-L1 to its receptor PD-1 on activated T cells inhibits antitumor immunity by counteracting T-cell–activating signals (39, 40). Ahn et al. showed that PD-1 plays a major role in CD8+ T-cell exhaustion during cancer and chronic infections (41). Therefore, we hypothesized that PD-L1 colocalization with CD8+ T cells would activate PD-1 and cause cell exhaustion. Our results show that the colocalization of PD-L1 on CD3+ T cells and CD8+ T cells is more strongly enhanced in advanced-stage tumors than in early-stage tumors (**Figure 3**).

Second, according to Kim et al., there is a growing consensus that CD4+ regulatory T cells play a crucial role in the suppression of the antitumor immune response and contribute to tumor escape from the host immune system (42). Our study also shows that CD4+ regulatory T cells are significantly more prevalent in advanced-stage dMMR CRCs than in early-stage dMMR CRCs (**Figure 2**). Considering the lack of validation and limited sample size, further investigation and experimentation with a larger sample size are strongly recommended. In conclusion, our study results highlight the possibility of heterogeneous distribution of T cells and PD-L1 colocalization in early-stage and advanced-stage dMMR CRCs.

PD-L1–positive cells were more frequently observed in advanced-stage tumors than in early-stage tumors in both epithelial and stromal regions (**Figure 1**), although this difference was not statistically significant. As mentioned in the literature, PD-L1 may counterbalance a vigorous immune microenvironment and promote dMMR tumor progression (39, 40). Therefore, our observation of a high number of PD-L1 colocalizing T cells, such as CD8+PD-L1+ cytotoxic T cells and CD8+PD-L1+ memory T cells, in advanced-stage CRC supports the use of ICIs, such as anti–PD-L1 drugs, in advanced-stage CRC. Further research should be conducted to investigate the possibility of the use of ICIs as a first-line therapy (3). PD-1–positive cells were more frequently seen in early-stage tumors than in advanced-stage tumors in epithelial regions. However, researchers have demonstrated that PD-1 expression level is not associated with the response rate of ICIs (1). In the case of macrophages, the proportion of PD-L1+ macrophages was increased in epithelial regions of advanced-stage dMMR tumors. Furthermore, among all immune cell phenotypes presented in **Table 2**, CD3+PD-1+ T cells were the only promising prognostic factor (**Figure 4**).

Exploring the clinicopathological features described in the literature, we observed that all dMMR tumors were associated with advanced age, location in the right colon, and poor differentiation, which is consistent with the results of previous studies. In advanced-stage dMMR tumors, a trend toward a sex ratio imbalance was observed (1:2 in favor of males). Among the 15% of CRCs with dMMR, approximately 3% were associated with Lynch syndrome, commonly with MLH1 and MSH2 mutations, and the other 12% were sporadic cases, predominantly caused by epigenetic silencing of MLH1 due to promoter hypermethylation (43, 44). The patients in our cohort were randomly chosen from resected cases; therefore, the proportions of Lynch syndrome cases and sporadic cases in our samples should be similar to those in patients with CRC in the general population. In early-stage dMMR CRC, we found that 3 of 24 patients had a BRAF mutation, and in advanced-stage dMMR CRC, 2 of 19 patients had a BRAF mutation, corresponding to normal percentages of BRAF mutation (**Supplementary Table 2**). However, considering a higher percentage of MSH2 and MSH6 expression loss in advanced-stage dMMR CRC, we hypothesized that Lynch syndrome would be found more often at the advanced stage. However, further exploration by means of the analysis of hypermethylation of the MLH1 promoter is needed. The mucinous histological subtype is the characteristic morphology of dMMR CRCs. It occurs in up to 40% of patients, according to previous reports (45); in our study, this subtype accounted for 29% of the advanced-stage tumors. Further mechanistic studies are needed to explain the variation in histological subtype across stages in dMMR CRCs.

Although our study was considerably strengthened by the synchronous analysis of multiple immune markers in the early and advanced stages of dMMR CRC, it also has limitations. The primary limitation is the finite number of samples in our patient cohort, which was because of the low proportion of patients with dMMR with advanced-stage cancers and few surgical opportunities to obtain specimens. We did not analyze detailed treatment patterns or all the classical features that were previously noted as potential prognostic factors because of the lack of related data. Furthermore, we hypothesized that PD-L1 colocalization with T cells would activate PD-1 and cause T-cell exhaustion, but we lacked functional studies or investigations of functional markers. Finally, we were not able to collect any information on the immunotherapeutic response, which leaves the predicted value of immune molecules unclear at this point.

In conclusion, we conducted a comprehensive evaluation of the immune microenvironment in the early and advanced stages of dMMR CRCs. Our results demonstrate that high numbers of CD8+ cytotoxic T cells and CD8+ memory T cells, which usually represent a cytotoxic function of the adaptive immune system, are associated with the transformation of the immune microenvironment from early-stage to advanced-stage dMMR CRCs. Furthermore, possibly enhanced inhibition factors, such as CD4+ regulatory T cells and upregulated PD-L1 colocalization of CD3+ T cells and CD8+ T cells, which were hypothesized to be exhausted, were found to be associated with the transformation from early-stage to advanced-stage dMMR CRCs. Furthermore, the immune cell population, such as CD3+PD-1+ cells, can be used to identify patients with dMMR with poor prognoses.

## Data Availability Statement

The original contributions presented in the study are included in the article/**Supplementary Material**. Further inquiries can be directed to the corresponding author.

## Ethics Statement

Written informed consent was obtained from the individual(s) for the publication of any potentially identifiable images or data included in this article.

## Author Contributions

Conception and design: DH. Development of the methodology: HY and YL. Analysis and interpretation: HY, XW, JQ, and MX. Writing and review: DH, HY, and JP. Study supervision: DH. All authors contributed to the article and approved the submitted version.

## Funding

This study is supported by grants from Shanghai Natural Science Foundation (19ZR1410200 to DH), National Natural Science Foundation of China (U1932145 to JP and 82002946 to YL), Science and Technology Commission of Shanghai Municipality (18401933402 to JP), Shanghai Sailing Program (19YF1409500 to YL), and “Chenguang Program” supported by Shanghai Education Development Foundation and Shanghai Municipal Education Commission (20CG08 to YL). The sponsors had no involvement in the study design, collection, analysis, interpretation of data, or writing of the report, nor in the decision to submit the paper for publication.

## Conflict of Interest

JQ is employed by Genecast Biotechnology Co., Ltd.

The remaining authors declare that the research was conducted in the absence of any commercial or financial relationship that could be construed as a potential conflict of interest.

## Publisher’s Note

All claims expressed in this article are solely those of the authors and do not necessarily represent those of their affiliated organizations, or those of the publisher, the editors and the reviewers. Any product that may be evaluated in this article, or claim that may be made by its manufacturer, is not guaranteed or endorsed by the publisher.

## Supplementary Material

Supplementary Figure 1 Pairwise comparisons of the contribution of immune cells between epithelial and stromal regions in patients with stage III–IV dMMR CRCs.

## References

[1] Le DT Uram JN Wang H Bartlett BR Kemberling H Eyring AD. Pd-1 Blockade in Tumors With Mismatch-Repair Deficiency. NEJM (2015) 372(26):2509–20. doi: 10.1056/NEJMoa1500596 PMC4481136 26028255

[2] Overman MJ McDermott R Leach JL Lonardi S Lenz HJ Morse MA. Nivolumab in Patients With Metastatic DNA Mismatch Repair-Deficient or Microsatellite Instability-High Colorectal Cancer (Checkmate 142): An Open-Label, Multicentre, Phase 2 Study. Lancet Oncol (2017) 18(9):1182–91. doi: 10.1016/S1470-2045(17)30422-9 PMC6207072 28734759

[3] André T Shiu KK Kim TW Jensen BV Jensen LH Punt C. Pembrolizumab in Microsatellite-Instability-High Advanced Colorectal Cancer. NEJM (2020) 383(23):2207–18. doi: 10.1056/NEJMoa2017699 33264544

[4] Leclerc J Vermaut C Buisine MP. Diagnosis of Lynch Syndrome and Strategies to Distinguish Lynch-Related Tumors From Sporadic MSI/dMMR Tumors. Cancers (Basel) (2021) 13(3):467. doi: 10.3390/cancers13030467 33530449 PMC7865821

[5] Turajlic S Litchfield K Xu H Rosenthal R McGranahan N Reading JL. Insertion-And-Deletion-Derived Tumour-Specific Neoantigens and the Immunogenic Phenotype: A Pan-Cancer Analysis. Lancet Oncol (2017) 18(8):1009–21. doi: 10.1016/s1470-2045(17)30516-8 28694034

[6] Willis JA Reyes-Uribe L Chang K. Immune Activation in Mismatch Repair-Deficient Carcinogenesis: More Than Just Mutational Rate. Clin Cancer Res (2020) 26(1):11–7. doi: 10.1158/1078-0432.ccr-18-0856 PMC6942620 31383734

[7] Lu C Guan J Lu S Jin Q Rousseau B Lu T. DNA Sensing in Mismatch Repair-Deficient Tumor Cells Is Essential for Anti-Tumor Immunity. Cancer Cell (2021) 39(1):96–108.e6. doi: 10.1016/j.ccell.2020.11.006 33338425 PMC9477183

[8] Germano G Lamba S Rospo G Barault L Magrì A Maione F. Inactivation of DNA Repair Triggers Neoantigen Generation and Impairs Tumour Growth. Nature (2017) 552(7683):116–20. doi: 10.1038/nature24673 29186113

[9] Koopman M Kortman GA Mekenkamp L Ligtenberg MJ Hoogerbrugge N Antonini NF. Deficient Mismatch Repair System in Patients With Sporadic Advanced Colorectal Cancer. Br J Cancer (2009) 100(2):266–73. doi: 10.1038/sj.bjc.6604867 PMC2634718 19165197

[10] Vilar E Tabernero J. Molecular Dissection of Microsatellite Instable Colorectal Cancer. Cancer Discov (2013) 3(5):502–11. doi: 10.1158/2159-8290.CD-12-0471 PMC3651752 23454900

[11] Jin Z Sanhueza CT Johnson B Nagorney DM Larson DW Mara KC. Outcome of Mismatch Repair-Deficient Metastatic Colorectal Cancer: The Mayo Clinic Experience. Oncologist (2018) 23(9):1083–91. doi: 10.1634/theoncologist.2017-0289 PMC6192616 29674439

[12] Toh JWT Phan K Reza F Chapuis P Spring KJ. Rate of Dissemination and Prognosis in Early and Advanced Stage Colorectal Cancer Based on Microsatellite Instability Status: Systematic Review and Meta-Analysis. Int J Colorec Dis (2021) 36(8):1573–96. doi: 10.1007/s00384-021-03874-1 33604737

[13] Chalabi M Fanchi LF Dijkstra KK Van den Berg JG Aalbers AG Sikorska K. Neoadjuvant Immunotherapy Leads to Pathological Responses in MMR-Proficient and MMR-Deficient Early-Stage Colon Cancers. Nat Med (2020) 26(4):566–76. doi: 10.1038/s41591-020-0805-8 32251400

[14] Petrelli F Ghidini M Ghidini A Tomasello G. Outcomes Following Immune Checkpoint Inhibitor Treatment of Patients With Microsatellite Instability-High Cancers: A Systematic Review and Meta-Analysis. JAMA Oncol (2020) 6(7):1068–71. doi: 10.1001/jamaoncol.2020.1046 PMC7226284 32407439

[15] Pagès F Kirilovsky A Mlecnik B Asslaber M Tosolini M Bindea G. *In Situ* Cytotoxic and Memory T Cells Predict Outcome in Patients With Early-Stage Colorectal Cancer. J Clin Oncol (2009) 27(35):5944–51. doi: 10.1200/jco.2008.19.6147 19858404

[16] Schrock AB Ouyang C Sandhu J Sokol E Jin D Ross JS. Tumor Mutational Burden Is Predictive of Response to Immune Checkpoint Inhibitors in MSI-High Metastatic Colorectal Cancer. Ann Oncol (2019) 30(7):1096–103. doi: 10.1093/annonc/mdz134 31038663

[17] Cristescu R Mogg R Ayers M Albright A Murphy E Yearley J. Pan-Tumor Genomic Biomarkers for Pd-1 Checkpoint Blockade-Based Immunotherapy. Science (2018) 362(6411):eaar3593. doi: 10.1126/science.aar3593 30309915 PMC6718162

[18] Cavalleri T Bianchi P Basso G Celesti G Grizzi F Bossi P. Combined Low Densities of Foxp3(+) and Cd3(+)tumor-Infiltrating Lymphocytes Identify Stage II Colorectal Cancer at High Risk of Progression. Cancer Immunol Res (2019) 7(5):751–8. doi: 10.1158/2326-6066.CIR-18-0661 30804005

[19] Yoon HH Shi Q Heying EN Muranyi A Bredno J Ough F. Intertumoral Heterogeneity of Cd3(+) and Cd8(+) T-Cell Densities in the Microenvironment of DNA Mismatch-Repair-Deficient Colon Cancers: Implications for Prognosis. Clin Cancer Res (2019) 25(1):125–33. doi: 10.1158/1078-0432.CCR-18-1984 PMC6320300 30301825

[20] Scheper W Kelderman S Fanchi LF Linnemann C Bendle G de Rooij MAJ. Low and Variable Tumor Reactivity of the Intratumoral Tcr Repertoire in Human Cancers. Nat Med (2019) 25(1):89–94. doi: 10.1038/s41591-018-0266-5 30510250

[21] Sahin IH Akce M Alese O Shaib W Lesinski GB El-Rayes B. Immune Checkpoint Inhibitors for the Treatment of MSI-H/MMR-D Colorectal Cancer and a Perspective on Resistance Mechanisms. Br J Cancer (2019) 121(10):809–18. doi: 10.1038/s41416-019-0599-y PMC6889302 31607751

[22] Olivo Pimentel V Yaromina A Marcus D Dubois LJ Lambin P. A Novel Co-Culture Assay to Assess Anti-Tumor Cd8(+) T Cell Cytotoxicity *via* Luminescence and Multicolor Flow Cytometry. J Immunol Methods (2020) 487:112899. doi: 10.1016/j.jim.2020.112899 33068606

[23] Fan A Wang B Wang X Nie Y Fan D Zhao X. Immunotherapy in Colorectal Cancer: Current Achievements and Future Perspective. Int J Biol Sci (2021) 17(14):3837–49. doi: 10.7150/ijbs.64077 PMC8495390 34671202

[24] Adamthwaite D Cooley MA. Cd8+ T-Cell Subsets Defined by Expression of Cd45 Isoforms Differ in Their Capacity to Produce Il-2, Ifn-Gamma and Tnf-Beta. Immunology (1994) 81(2):253–60. PMC1422313 7908892

[25] Hameed A Hruban RH Gage W Pettis G Fox WM 3rd. Immunohistochemical Expression of Cd68 Antigen in Human Peripheral Blood T Cells. Hum Pathol (1994) 25(9):872–6. doi: 10.1016/0046-8177(94)90005-1 8088761

[26] Klinge U Dievernich A Tolba R Klosterhalfen B Davies L. Cd68+ Macrophages as Crucial Components of the Foreign Body Reaction Demonstrate an Unconventional Pattern of Functional Markers Quantified by Analysis With Double Fluorescence Staining. J BioMed Mater Res (2020) 108(8):3134–46. doi: 10.1002/jbm.b.34639 32475069

[27] Kunisch E Fuhrmann R Roth A Winter R Lungershausen W Kinne RW. Macrophage Specificity of Three Anti-Cd68 Monoclonal Antibodies (Kp1, Ebm11, and Pgm1) Widely Used for Immunohistochemistry and Flow Cytometry. Ann Rheum Dis (2004) 63(7):774–84. doi: 10.1136/ard.2003.013029 PMC1755048 15194571

[28] Wang M Windgassen D Papoutsakis ET. Comparative Analysis of Transcriptional Profiling of Cd3+, Cd4+ and Cd8+ T Cells Identifies Novel Immune Response Players in T-Cell Activation. BMC Genom (2008) 9:225. doi: 10.1186/1471-2164-9-225 PMC2396644 18485203

[29] Gorris MAJ Halilovic A Rabold K van Duffelen A Wickramasinghe IN Verweij D. Eight-Color Multiplex Immunohistochemistry for Simultaneous Detection of Multiple Immune Checkpoint Molecules Within the Tumor Microenvironment. J Immunol (2018) 200(1):347–54. doi: 10.4049/jimmunol.1701262 29141863

[30] Ge P Wang W Li L Zhang G Gao Z Tang Z. Profiles of Immune Cell Infiltration and Immune-Related Genes in the Tumor Microenvironment of Colorectal Cancer. BioMed Pharmacother (2019) 118:109228. doi: 10.1016/j.biopha.2019.109228 31351430

[31] Schmitt M Greten FR. The Inflammatory Pathogenesis of Colorectal Cancer. Nat Rev Immunol (2021) 21(10):653–67. doi: 10.1038/s41577-021-00534-x 33911231

[32] Beyaz S Mana MD Roper J Kedrin D Saadatpour A Hong SJ. High-Fat Diet Enhances Stemness and Tumorigenicity of Intestinal Progenitors. Nature (2016) 531(7592):53–8. doi: 10.1038/nature17173 PMC4846772 26935695

[33] Schmitt M Schewe M Sacchetti A Feijtel D van de Geer WS Teeuwssen M. Paneth Cells Respond to Inflammation and Contribute to Tissue Regeneration by Acquiring Stem-Like Features Through Scf/C-Kit Signaling. Cell Rep (2018) 24(9):2312–28.e7. doi: 10.1016/j.celrep.2018.07.085 30157426

[34] Pesic M Greten FR. Inflammation and Cancer: Tissue Regeneration Gone Awry. Curr Opin Cell Biol (2016) 43:55–61. doi: 10.1016/j.ceb.2016.07.010 27521599

[35] Greten FR Grivennikov SI. Inflammation and Cancer: Triggers, Mechanisms, and Consequences. Immunity (2019) 51(1):27–41. doi: 10.1016/j.immuni.2019.06.025 31315034 PMC6831096

[36] Grivennikov SI Greten FR Karin M. Immunity, Inflammation, and Cancer. Cell (2010) 140(6):883–99. doi: 10.1016/j.cell.2010.01.025 PMC2866629 20303878

[37] Han J Khatwani N Searles TG Turk MJ Angeles CV. Memory Cd8(+) T-Cell Responses to Cancer. Semin Immunol (2020) 49:101435. doi: 10.1016/j.smim.2020.101435 33272898 PMC7738415

[38] He QF Xu Y Li J Huang ZM Li XH Wang X. Cd8+ T-Cell Exhaustion in Cancer: Mechanisms and New Area for Cancer Immunotherapy. Brief Funct Genom (2019) 18(2):99–106. doi: 10.1093/bfgp/ely006 29554204

[39] Poggio M Hu T Pai CC Chu B Belair CD Chang A. Suppression of Exosomal Pd-L1 Induces Systemic Anti-Tumor Immunity and Memory. Cell (2019) 177(2):414–27.e13. doi: 10.1016/j.cell.2019.02.016 30951669 PMC6499401

[40] Dammeijer F van Gulijk M Mulder EE Lukkes M Klaase L van den Bosch T. The Pd-1/Pd-L1-Checkpoint Restrains T Cell Immunity in Tumor-Draining Lymph Nodes. Cancer Cell (2020) 38(5):685–700.e8. doi: 10.1016/j.ccell.2020.09.001 33007259

[41] Ahn E Araki K Hashimoto M Li W Riley JL Cheung J. Role of Pd-1 During Effector Cd8 T Cell Differentiation. PNAS (2018) 115(18):4749–54. doi: 10.1073/pnas.1718217115 PMC5939075 29654146

[42] Kim JH Kim BS Lee SK. Regulatory T Cells in Tumor Microenvironment and Approach for Anticancer Immunotherapy. Immune Netw (2020) 20(1):e4. doi: 10.4110/in.2020.20.e4 32158592 PMC7049587

[43] Boland CR Goel A. Microsatellite Instability in Colorectal Cancer. Gastroenterology (2010) 138(6):2073–87.e3. doi: 10.1053/j.gastro.2009.12.064 20420947 PMC3037515

[44] Jiricny J. The Multifaceted Mismatch-Repair System. Nat Rev Mol Cell Biol (2006) 7(5):335–46. doi: 10.1038/nrm1907 16612326

[45] Andrici J Farzin M Sioson L Clarkson A Watson N Toon CW. Mismatch Repair Deficiency as a Prognostic Factor in Mucinous Colorectal Cancer. Mod Pathol (2016) 29(3):266–74. doi: 10.1038/modpathol.2015.159 26769140

