# Supplementary figures and images for: The Alteration of T-Cell Heterogeneity and PD-L1 Colocalization During dMMR Colorectal Cancer Progression Defined by Multiplex Immunohistochemistry
Source: Front Oncol. 2022 May 20;12:867658. doi: 10.3389/fonc.2022.867658 (PMC9163547; doi:10.3389/fonc.2022.867658)

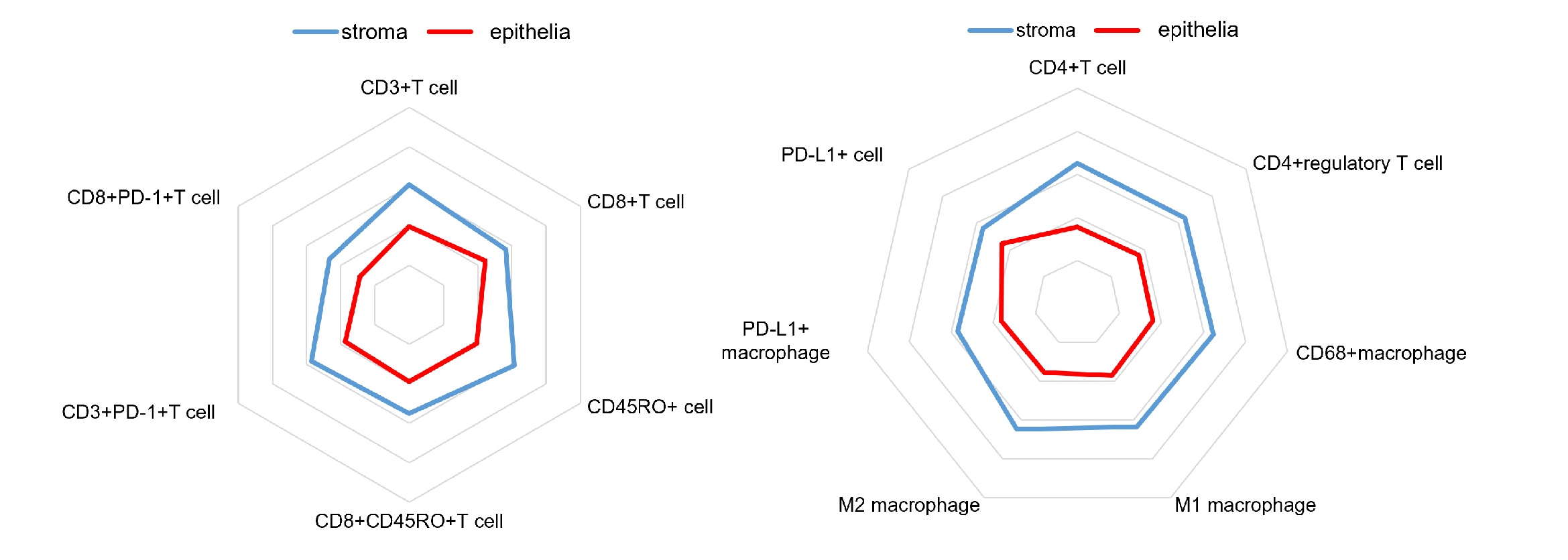

Supplement: Supplementary Figure 1 — Pairwise comparisons of the contribution of immune cells between epithelial and stromal regions in patients with stage III–IV dMMR CRCs. [file Image_1.jpg]
