# Supplementary material for: The Alteration of T-Cell Heterogeneity and PD-L1 Colocalization During dMMR Colorectal Cancer Progression Defined by Multiplex Immunohistochemistry
Source: Front Oncol. 2022 May 20;12:867658. doi: 10.3389/fonc.2022.867658 (PMC9163547; doi:10.3389/fonc.2022.867658)
Supplement: Supplementary file 2 [file Table_1.docx]

# Supplementary Table

**Supplementary Table 1. Immunohistochemical multiplex staining protocol**

| **Primary antibody** | **Provider** | **Clone** | **Catalogue number** | **Concentration** |
| --- | --- | --- | --- | --- |
| **panel1** |  |  |  |  |
| CD3 | Zsbio | UMAB54 | ZM-0417 | 1:50 |
| CD8 | Zsbio | EP334 | ZA-0508 | 1:100 |
| CD45RO | Zsbio | UCH-L1 | ZM-0055 | 1:200 |
| PD-1 | Zsbio | UMAB199 | ZM-0381 | 1:100 |
| PD-L1 | Roche | SP142 | 740-4859 | 1:25 |
| **panel2** |  |  |  |  |
| CD4 | Zsbio | UMAB64 | ZM-0418 | 1:1 |
| FoxP3 | Abcam | 236A/E7 | Ab20034 | 1:400 |
| PD-L1 | Roche | SP142 | 740-4859 | 1:25 |
| CD68 | Zsbio | KP1 | ZM-0060 | 1:500 |
| CD163 | Zsbio | 10D6 | ZM-0428 | 1:100 |

**Supplementary Table 2. Patients’ characteristics**

| **Variable** | **Advanced-stage dMMR CRCs （Stage Ⅲ-Ⅳ）** | | **Early-stage dMMR CRCs**  **（Stage Ⅱ）** | |
| --- | --- | --- | --- | --- |
|  | No. of Patients (n=59) | % | No. of Patients (n=24) | % |
| **Gender** |  |  |  |  |
| male | 39 | 66 | 13 | 54 |
| female | 20 | 34 | 11 | 46 |
| **Age (years)** |  |  |  |  |
| ＜65y | 46 | 78 | 15 | 62 |
| ≥65y | 13 | 22 | 9 | 38 |
| **Tumor location** |  |  |  |  |
| right hemicolon | 33 | 56 | 16 | 67 |
| left hemicolon | 16 | 27 | 3 | 13 |
| rectum | 10 | 17 | 5 | 21 |
| **Tumor size** |  |  |  |  |
| ≤5cm | 31 | 53 | - |  |
| ＞5cm | 28 | 47 | - |  |
| **Tumor grade** |  |  |  |  |
| 3 | 31 | 52 | 11 | 46 |
| 2 | 28 | 48 | 11 | 46 |
| 1 | 0 | 0 | 2 | 8 |
| **Histological subtype** |  |  |  |  |
| Mucinous | 17 | 29 | 10 | 42 |
| Other | 42 | 71 | 14 | 58 |
| **MMR status** |  |  |  |  |
| Loss of MLH1 and PMS2 | 35 | 60 | 12 | 50 |
| Loss of MSH2 and MSH6 | 16 | 27 | 4 | 17 |
| Isolated loss of PMS2 | 2 | 3 | 2 | 8 |
| Isolated loss of MSH6 | 6 | 10 | 6 | 25 |
| **Tumor stage** |  |  |  |  |
| T1 | 0 | 0 | 0 | 0 |
| T2 | 4 | 7 | 0 | 0 |
| T3 | 40 | 68 | 10 | 42 |
| T4 | 15 | 35 | 14 | 58 |
| **Regional LNs metastasis** |  |  |  |  |
| N0 | 3 | 5 | 0 | - |
| N1 | 33 | 56 | 0 | - |
| N2 | 23 | 39 | 0 | - |
| **Distant metastasis** |  |  |  |  |
| M0 | 46 | 78 | 0 | - |
| M1 | 13 | 22 | 0 | - |
| **AJCC^#^ stage** |  |  |  |  |
| II | 0 | 0 | 24 | 100 |
| III | 46 | 78 | 0 | 0 |
| IV | 13 | 22 | 0 | 0 |
| **Vascular invasion** |  |  |  |  |
| negative | 27 | 46 | 21 | 88 |
| positive | 32 | 54 | 3 | 12 |
| **Perineural invasion** |  |  |  |  |
| negative | 41 | 69 | 17 | 71 |
| positive | 18 | 31 | 7 | 29 |
| **LNR^*^** |  |  |  |  |
| ＜0.2 | 38 | 64 | - | - |
| ≥0.2 | 21 | 36 | - | - |
| **Plasma CEA^†^ (ng/ml)** |  |  |  |  |
| ＜5 | 29 | 49 | 16 | 67 |
| ≥5 | 30 | 51 | 8 | 33 |
| **Chemotherapy** |  |  |  |  |
| yes | 55 | 93 | 16 | 67 |
| no | 4 | 7 | 8 | 33 |
| **Radiotherapy** |  |  |  |  |
| yes | 2 | 3 | 2 | 12 |
| no | 57 | 97 | 21 | 88 |
| **BRAF** |  |  |  |  |
| mut | 2 | 11 | 3 | 13 |
| wt | 17 | 89 | 21 | 87 |

#: AJCC: American Joint Committee on Cancer, 8th;

*: LNR: Lymph node ratio between metastatic and examined lymph nodes

†: CEA: Carcinoembryonic antigen
